# *Ralstonia solanacearum RSp0194* Encodes a Novel 3-Keto-Acyl Carrier Protein Synthase III

**DOI:** 10.1371/journal.pone.0136261

**Published:** 2015-08-25

**Authors:** Ya-Hui Mao, Jin-Cheng Ma, Feng Li, Zhe Hu, Hai-Hong Wang

**Affiliations:** Guangdong Provincial Key Laboratory of Protein Function and Regulation in Agricultural Organisms, College of Life Sciences, South China Agricultural University, Guangzhou, Guangdong 510642, China; University of Illinois at Urbana-Champaign, UNITED STATES

## Abstract

Fatty acid synthesis (FAS), a primary metabolic pathway, is essential for survival of bacteria. *Ralstonia solanacearum*, a β-proteobacteria member, causes a bacterial wilt affecting more than 200 plant species, including many economically important plants. However, thus far, the fatty acid biosynthesis pathway of *R*. *solanacearum* has not been well studied. In this study, we characterized two forms of 3-keto-ACP synthase III, RsFabH and RsFabW, in *R*. *solanacearum*. RsFabH, the homologue of *Escherichia coli* FabH, encoded by the chromosomal *RSc1050* gene, catalyzes the condensation of acetyl-CoA with malonyl-ACP in the initiation steps of fatty acid biosynthesis *in vitro*. The *RsfabH* mutant lost *de novo* fatty acid synthetic ability, and grows in medium containing free fatty acids. RsFabW, a homologue of *Pseudomonas aeruginosa* PA3286, encoded by a megaplasmid gene, *RSp0194*, condenses acyl-CoA (C_2_-CoA to C_10_-CoA) with malonyl-ACP to produce 3-keto-acyl-ACP *in vitro*. Although the *RsfabW* mutant was viable, *RsfabW* was responsible for *RsfabH* mutant growth on medium containing free fatty acids. Our results also showed that RsFabW could condense acyl-ACP (C_4_-ACP to C_8_-ACP) with malonyl-ACP, to produce 3-keto-acyl-ACP *in vitro*, which implies that RsFabW plays a special role in fatty acid synthesis of *R*. *solanacearum*. All of these data confirm that *R*. *solanacearum* not only utilizes acetyl-CoA, but also, utilizes medium-chain acyl-CoAs or acyl-ACPs as primers to initiate fatty acid synthesis.

## Introduction

**Fig 1:**
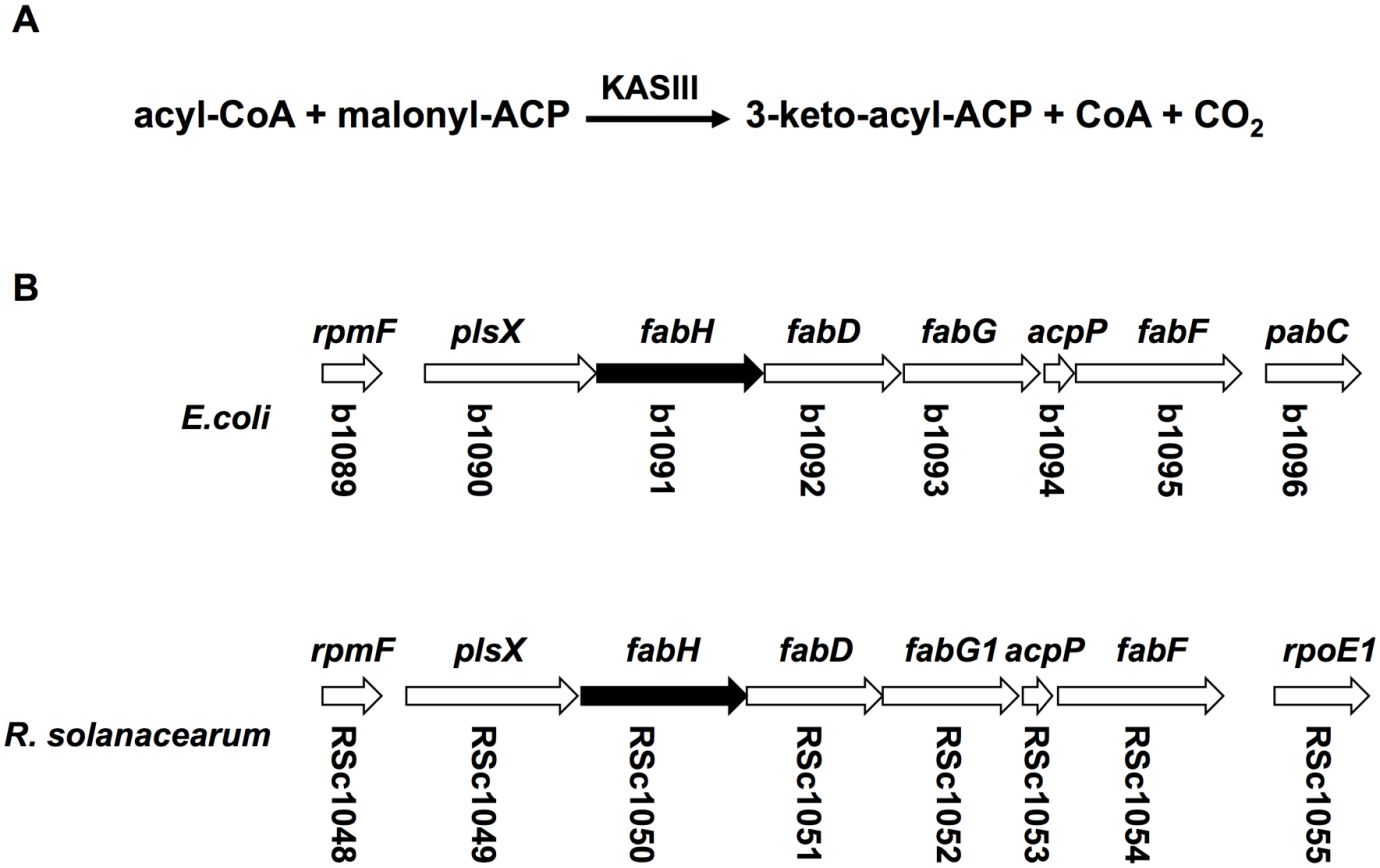
The 3-ketoacyl-ACP synthase III reaction, organization of the *R*. *solanacearum* fatty acid biosynthesis gene clusters and alignment of *R*. *solanacearum* FabH and RSp0194 with *E*. *coli* FabH or *P*. *aeruginosa* PA3286. (A) The reaction catalyzed by 3-ketoacyl-ACP synthase III. (B) Organization of the *E*.*coli* and *R*. *solanacearum* fatty acid biosynthesis gene clusters. The thick arrows indicate the relative sizes of the genes. The numbers below the arrows indicate the gene designations in the *E*. *coli* or *R*. *solanacearum* GeneBank database. (C) and (D) Alignments of *R*. *solanacearum* FabH with *E*. *coli* FabH (C) or *R*. *solanacearum* RSp0194 with *P*. *aeruginosa* PA3286 (D). Rs, Ec, and Pa are denoted as *R*. *solanacearum*, *E*. *coli*, and *P*. *aeruginosa*, respectively. The catalytic triad (Cys-His-Asn) are highlighted by asterisks. The alignment was done with Clustal W, based on identical residues.

Fatty acid synthesis (FAS) is a primary bacterial metabolic pathway. Fatty acids are not only bacterial cell components (membrane phospholipids, lipoproteins, and lipoglycans) [1,2,3,4], but also intermediates, used to synthesize other end products, such as cofactors (lipoate [5] and biotin [6,7]), quorum sensing [QS] signal molecules [8], and energy storage components [9]. In most bacteria, fatty acid synthase (FAS II) is composed of a series of small soluble proteins, and each enzyme, encoded by a separate gene, carries out a different catalytic step in the pathway [1,2,4]. In the fatty acid biosynthetic pathway of bacteria, 3-ketoacyl-ACP synthase (KAS) condenses acyl-acyl carrier protein (acyl-ACP) or acyl coenzyme A (acyl-CoA) with malonyl-ACP to produce 3-ketoacyl-ACP. Most bacteria contain at least two classes of KAS enzymes. KAS III class enzymes, which have a Cys-His-Asn active site triad, are responsible for the initiation of the fatty acid chains (Fig 1A), whereas the KAS I/II classes of condensing enzymes have a Cys-His-His catalytic triad, and catalyze synthesis of long acyl chains in the elongation steps [4,10,11]. In general, 3-ketoacyl-ACP synthase III (FabH) has two roles in bacterial fatty acid biosynthesis. First, as the initiator of elongation, this enzyme determines the amount of fatty acids that will be produced by the pathway, and is therefore subject to stringent feedback regulation by long-chain acyl-ACPs [12]. Second, the substrate specificity of this enzyme is a major determining factor for membrane fatty acid composition of bacteria [10,13]. In *Escherichia coli*, FabH prefers to use acetyl-CoA as a primer, leading to the only production of straight-chain fatty acids in this organism [12,14]. In *Listeria monocytogenes* and many gram-positive bacteria, the FabH proteins are highly selective for branched-chain acyl-CoA as substrates, and primarily *iso*- and *anteiso*-branched chain fatty acids are produced in these bacteria [15,16]. In *Mycobacterium tuberculosis*, the type II fatty acid biosynthesis pathway is employed to produce very long-chain mycolic acids, and accordingly the MtFabH enzyme prefers long-chain acyl-CoA substrates [10]. Recently, Yuan *et al*. identified two novel types of 3-ketoacyl-ACP synthase III in *Pseudomonas aeruginosa*, FabY and PA3286 [9,17]. FabY, defined as a novel class of 3-ketoacyl synthase KASI/II domain condensation enzymes, possesses a typical Cys-His-His catalytic triad and catalyzes the condensation of acetyl-CoA with malonyl-ACP in initiation steps of fatty acid biosynthesis of *P*. *aeruginosa* [17]. PA3286, which belongs to the 3-ketoacyl-acyl carrier protein synthase III family, condenses malonyl-ACP with octanoyl-CoA shunting from the fatty acid β-oxidation degradation cycle, to produce 3-ketodecanoyl-ACP, which is a key intermediate common for synthesis of saturated fatty acids (SFA), unsaturated fatty acids (UFA) and lipopolysaccharides (LPS) [9].

*Ralstonia solanacearum*, a soil-borne, destructive plant pathogen, causes a bacterial wilt that affects many economically important plants such as tomato (*Lycopersicon esculentum*), potato (*Solanum tuberosum*), tobacco (*Nicotiana tabacum*), and banana (*Musa acuminata*) [18,19]. *R*. *solanacearum* is known to utilize a complex regulatory pathway to activate production of the major virulence factors. Two signal transition pathways using *hrp* and *phc*, have been reported [20]. In the *phc* pathway, a LysR-type transcriptional regulator, PhcA, plays a central role in response to cell density by using a quorum-sensing mechanism that involves the specific signal molecule, 3-hydroxypalmitic acid methyl ester (3-OH-PAME). It has been demonstrated that 3-OH-PAME is synthesized by PhcB, which is thought to convert 3-hydroxypalmitoyl-ACP to 3-hydroxypalmitoyl methyl ester [21]. The 3-hydroxypalmitoyl-ACP comes from the bacterial fatty acid synthesis pathway. This indicates that novel antimicrobials targeting the enzymes in the *R*. *solanacearum* fatty acid synthesis pathway would interfere with quorum sensing of *R*. *solanacearum* and this could be helpful to control the bacterial wilt caused by *R*. *solanacearum*.

Although fatty acid analyses of total membranes of *R*. *solanacearum* demonstrated typical fatty acids found in many proteobacteria, primarily palmitic (C_16:0_), palmitoleic (C_16:1_), and *cis*-vaccenic (C_18:1_) acids [21,22], little is known about the *R*. *solanacearum* fatty acid synthesis pathway. Cheng *et al*. have characterized five *R*. *solanacearum* putative 3-ketoacyl-ACP synthase homologues (RSc1054, RSp0358, RSp0357, RSp0361, and RSc0427) in *R*. *solanacearum*, and found that only RSc1054 possesses long-chain 3-ketoacyl-ACP synthase activity and is required for fatty acid synthesis [23]. Recently, our laboratory has studied two 3-ketoacyl-ACP reductase homologues (RSc1052 and RSp0359) in *R*. *solanacearum* and the data suggested both enzymes are active in fatty acid synthesis but function differently in determining the profile of cellular fatty acids (unpublished data).

In this study, we identified two forms of 3-ketoacyl-ACP synthase III, FabH and RSp0194, in *R*. *solanacearum*. Results have shown that RsFabH, similar to *E*. *coli* FabH, catalyzes the condensation of acetyl-CoA with malonyl-ACP in the initiation steps of fatty acid biosynthesis, and RSp0194, like *P*. *aeruginosa* PA3286, prefers to condense medium-chain acyl-CoAs (such as octanoyl-CoA or decanoyl-CoA) substrates with malonyl-ACP. However, it is also able to condense short-chain acyl-ACPs (such as butanoyl-ACP, hexanoyl-ACP, and octanoyl-ACP) with malonyl-ACP in elongation steps to produce decanoyl-ACP. This implies RSp0194 plays a unique role in fatty acid synthesis of *R*. *solanacearum*.

## Materials and Methods

### Materials

Malonyl coenzyme A (malonyl-CoA), acyl-CoAs, fatty acids, NADH, NADPH, and antibiotics were purchased from Sigma-Aldrich (St. Louis, MO, USA); Takara Biotechnology Co., Ltd. (Dalian, China) provided molecular biology reagents; the Ni^2+^-agarose columns were purchased from Invitrogen (Shanghai, China); American Radiolabeled Chemicals, Inc. (St. Louis, MO, USA) provided sodium [1-^14^C] acetate (specific activity, 50 mCi/mM); and Bio-Rad (Hercules, CA, USA) provided the Quick Start Bradford dye reagent. All other reagents were of the highest available quality.

### Bacterial strains, plasmids, and growth conditions

Strains and plasmids used in this study are listed in S1 Table. LB medium was used as the rich medium for *E*. *coli* strains. *R*. *solanacearum* strains were cultured at 30°C in BG broth (1% Bacto peptone, 0.1% casamino acids, 0.1% yeast extract, and 0.5% glucose) or BG agar (BG broth plus 1.5% agar) [23]. Antibiotics were used at the following concentrations: 10 μg/mL gentamycin (for *E*. *coli*) or 30 μg/mL (for *R*. *solanacearum*); 30 μg/mL kanamycin sulfate; 100 μg/mL sodium ampicillin, and 30 μg/mL chloramphenicol. Isopropyl-β-D-thiogalactoside (IPTG) was used at a final concentration of 1 mM.

### Recombinant DNA techniques and construction of plasmids

To clone the *R*. *solanacearum fabH* (*RSc1050*) and *fabW* (*RSp0194*) genes, genomic DNA, which was extracted from strain GMI1000, was used for PCR amplification with *Pfu* DNA polymerase, using the primers listed in S2 Table. The PCR products were inserted into the T-vector plasmid, pMD19, to produce plasmids pYH1 (*RsfabH*) and pYH12 (*RsfabW*). The *RsfabH* and *RsfabW* genes were confirmed by nucleotide sequencing performed by Shanghai Sangon Inc. (Shanghai, China). The pYH1 and pYH12 plasmids were digested with restriction endonucleases as described in S2 Table, and then inserted into the vector pSRK-Gm [24] to yield plasmid pYH3 (pSRK-*RsfabH*) or pYH6 (pSRK-*RsfabW*), or inserted into pET28 (b) to obtain plasmids pYH7 (pET-*RsfabH*) or pYH9 (pET-*RsfabW*). Plasmids pYH2 (*EcfabH*), pYH4 (pSRK-*EcfabH*), pYH8 (pET-*EcfabH*), and pYH5 (pSRK-*aasS*) were also constructed.

To create an unmarked deletion mutant of *RsfabH*, the 500 base pair (bp) DNA fragments flanking up or down the *fabH* gene were amplified with *Pfu* DNA polymerase using *R*. *solanacearum* genomic DNA as the template, and either RsfabH Up EcoRI and RsfabH Up XbaI (for Up *fabH*), or RsfabH Dn XbaI and RsfabH Dn HindIII (for Down *fabH*) as primers (S2 Table), respectively. The products of these PCR reactions were purified and overlapping PCR was carried out using RsfabH Up EcoRI and RsfabH Dn HindIII as the primers. The resulting 1,000 bp DNA fragment was digested with EcoRI and HindIII and inserted between the same sites of pK18mobscaB [25] to yield pYH10. Using the same methods, an *RsfabW* deletion plasmid pYH11 was also constructed.

### Deletion of the *RsfabH* and *RsfabW* genes

*E*. *coli* strain S17-1 carrying plasmid pYH10 or pYH11 was conjugated with *R*. *solanacearum* GMI1000 on BG plates for 24 h at 30°C. After appropriate dilutions, the cultures were spread onto BG plates containing chloramphenicol plus kanamycin to select for integration of the plasmid into the genome of GMI1000. Several colonies were inoculated into BG medium (for selection of the Δ*fabW* strain) or BG medium containing 0.01% octanoic acid (for selection of the *ΔfabH* strain), at 30°C for 48 h. After appropriate dilutions, the cultures were spread onto BG plates containing 10% sucrose and octanoic acid. Colony PCR utilizing the primers listed in S2 Table was carried out to screen colonies sensitive to kanamycin. We successfully obtained *RsfabH* deletion strain RsmH and *RsfabW* deletion strain RsmW. To obtain the Δ*fabH* Δ*fabW* double deletion strain, the *Vibrio harveyi* AasS expression plasmid pYH5 (pSRK-*aasS*) was first introduced into strain RsmH (Δ*fabH*). Then, using the same method as described above, the double deletion mutant Δ*fabH* Δ*fabW*/ pSRK-*aasS* strain, RsmD, was obtained.

### Assays of *de novo* fatty acid synthesis

To examine the function of *fabH* in *R*. *solanacearum* fatty acid synthesis, *de novo* fatty acid synthesis of RsmH (Δ*fabH*) strain was measured by sodium [1-^14^C] acetate incorporation into membrane phospholipids. The RsmH (Δ*fabH*) strain was grown at 30°C in BG supplemented with 0.01% octanoic acid for 36 h. The cells were harvested and washed three times with fresh BG to remove unincorporated fatty acids. The cells were transferred into 10 mL BG medium, and then 5 μL of sodium [1-^14^C] acetate was added. The culture was incubated for an additional 4 h. Labeled fatty acids were extracted, analyzed by thin layer chromatography (TLC), and quantitated by phosphorimaging [26].

### Expression and purification of His-tagged proteins

Plasmids pYH7 (*RsfabH)* and pYH9 (*RsfabW*) were each transformed into BL21 (DE3) cells. To assay for protein solubility, the expression of these two proteins was first analyzed by SDS-PAGE (data was not shown). The RsFabH and RsFabW were purified by nickel-chelate chromatography as described previously [27,28]. We also purified the *E*. *coli* FabD, FabH, FabG, FabA, FabI, and FabB proteins, *Vibrio harveyi* acyl-ACP synthetase (AasS), and the *E*. *coli* holo-acyl carrier protein (ACP), as described previously [27,28].

### Assay of RsFabH and RsFabW activity *in vitro*

To test whether FabW condenses acyl-ACPs with malonyl-ACP, the acyl-ACPs (C_6_-ACP to C_10_-ACP) were synthesized from the fatty acids, ATP, and *E*. *coli* holo-ACP by *V*. *harveyi* acyl-ACP synthetase (AasS), as described previously [29,30]. The function of RsFabH or RsFabW in the initiation of fatty acid synthesis was tested in reaction mixtures containing 0.1 M sodium phosphate (pH 7.0), 0.1 μg each of EcFabD, EcFabG, EcFabZ, and EcFabI, 50 μM NADH, 50 μM NADPH, 1 mM β-mercaptoethanol, 100 μM acyl-CoA (or acetyl-CoA, or acyl-ACP), 100 μM malonyl-CoA, and 50 μM holo-ACP in a final volume of 40 μL. The reactions were initiated by addition of 0.1μg 3-ketoacyl-ACP synthase III (EcFabH, RsFabH, or RsFabW). After incubation for 2 h at 37°C, the reaction products were resolved by conformationally sensitive gel electrophoresis as described previously [29,30]. To verify the products of RsFabW catalyzed reaction, 500 μL of the above reaction mixture was used, and acyl-ACPs derivatives were purified according to the method of Zhao *et al*. [31]. Their molecular masses were determined by matrix-assisted laser desorption/ionization (MALDI)-time-of-flight (TOF)-mass spectrometry (MS) (Bruker Autoflex III; Freemont, CA, USA) according to the methods described previously [32].

### Enzyme assay

The 3-ketoacyl-ACP synthase III activities of RsFabH or RsFabW were determined by monitoring the rate of oxidation of NADPH at 340 nm using an extinction coefficient of 6,220 M^-1^. The reaction mixtures for activity assays contained 200 μM NADPH, 0.1 μg of the purified native EcFabG (*E*.*coli* 3-ketoacyl-ACP reductase) and EcFabD (*E*.*coli* malonyl-CoA:ACP transcylase), 100 μM holo-ACP, 500 μM malonyl-CoA, 500 μM acyl-CoA (C_2_-CoA to C_10_-CoA), and 0.1 M sodium phosphate buffer (pH 6.4). The reactions were initiated by addition of 0.1 μg of 3-ketoacyl-ACP synthase III (RsFabH or RsFabW).

### Cell-free extract preparation

Cell extracts of *R*. *solanacearum* strains were prepared from exponentially growing cells. Cell cultures were harvested by centrifugation and then re-suspended in lysis buffer (0.1 M sodium phosphate, pH 7.5, 5 mM β-mercaptoethanol, and 1 mM EDTA). Cell lysates were prepared by passing the cultures three times through a French pressure cell, then they were centrifuged for 1 h at 260,000 × g, and the supernatants were dialyzed against lysis buffer for 24 h and saved as cell extracts.

## Results

### *R*. *solanacearum* genes encode two different 3-ketoacyl-acyl carrier protein synthase III homologues

To identify putative 3-ketoacyl-ACP synthase III encoding genes in *R*. *solanacearum*, the sequences of FabH from *E*. *coli*, and FabY and PA3286 [9,17] from *P*. *aeruginosa* were used as query sequences for a BLAST analysis of the *R*. *solanacearum* GMI1000 genome [19]. The chromosomal *RSc1050* gene (named *RsfabH* in this study) is annotated as encoding an *E*. *coli* FabH homologue. The *RSp0194* gene (named *RsfabW* in this study), located on a megaplasmid, encodes a strong homologue of *P*. *aeruginosa* PA3286. Alignment of *R*. *solanacearum* FabH and *E*. *coli* FabH showed that RsFabH is 53.4% identical to *E*. *coli* FabH (Fig 1C). Moreover, *RsfabH* is located within a fatty acid synthesis gene cluster (*plsX*, *fabH*, *fabD*, *fabG1*, *acpP*, and *fabF*) (Fig 1B) and it has been shown that the gene cluster encoded FabF proteins have roles in *R*. *solanacearum* fatty acid biosynthesis [23]. RsFabW is 60.9% identical to PA3286, and more importantly, contains the Cys-His-Asn catalytic active triad like the 3-ketoacyl-ACP synthase III family of proteins (Fig 1D). These alignments suggest that the *R*. *solanacearum* genome encodes at least two different 3-ketoacyl-ACP synthase III homologues.

### Properties of *R*. *solanacearum ΔfabH* and *ΔfabW* deletion mutant strains

To identify the physiological functions of the two KASIIIs in *R*. *solanacearum* fatty acid biosynthesis, knockout strains for deletion of the *RsfabH* or *RsfabW* genes were made by allelic replacement. Two suicide plasmids, pYH10 and pYH11, were constructed, and then introduced into strain GMI1000 by conjugation (see Materials and Methods). The conjugants were selected on BG medium containing kanamycin. Cultures from conjugants were then plated onto BG medium containing sucrose in order to select for the loss of the suicide plasmid sequences from the GMI1000 genome (for selection of the *RsfabH* deletion strain, BG medium also contained octanoic acid). The colonies that were sensitive to kanamycin and resistant to sucrose were assayed by PCR analysis using the primers listed in S2 Table (data not shown). Two deletion mutant strains were obtained: RsmH (*ΔfabH*) and RsmW (*ΔfabW*).

**Fig 2:**
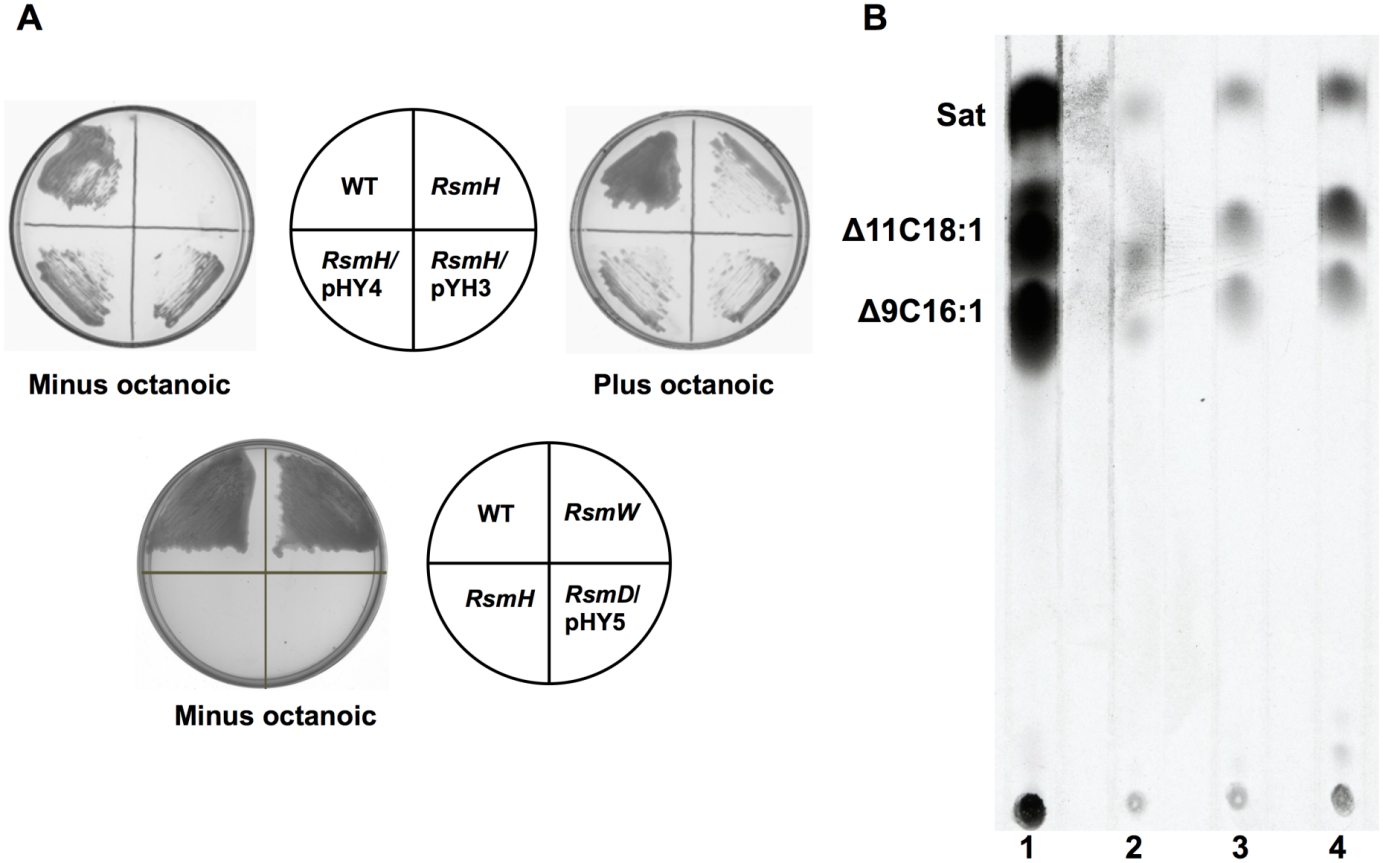
Growth of *R*. *solanacearum fabH* or *fabW* mutants on BG medium and fatty acid synthesis of *R*. *solanacearum fabH*. (A) Growth of *R*. *solanacearum fabH* or *fabW* mutants on BG medium in the absence or presence of octanoic acid. (B) Argentation thin-layer chromatographic analysis of sodium [1-^14^C] acetate-labeled *R*. *solanacearum fabH* mutant as described in Materials and Methods. Sat, saturated fatty acid esters; Δ9C16: 1, methyl ester of *cis*-9-hexadecenoic acid; Δ11C18: 1, methyl ester of *cis*-11-octadecenoic acid. Lane 1 is the methyl esters of wild-type *R*. *solanacearum* GMI1000, lane 2 is the methyl esters of *R*. *solanacearum fabH* mutant strain RsmH, lane 3 is the methyl esters of *R*. *solanacearum fabH* mutant strain RsmH carrying *R*. *solanacearum fabH* encoded plasmid pYH3, and lane 4 is the methyl esters of *R*. *solanacearum fabH* mutant strain RsmH carrying *E*. *coli fabH* encoded plasmid pYH4.

We first tested the growth of the RsmH and RsmW strains on BG medium. Strain RsmH failed to grow on BG medium except in the presence of octanoic acid, whereas strain RsmW grew on BG medium in the absence of octanoic acid, as did the wild type strain GMI1000 (Fig 2A). When either *E*. *coli fabH* or *R*. *solanacearum fabH* was expressed from plasmid pSRK-Gm in strain RsmH, growth on BG medium in the absence of octanoic acid was restored (Fig 2A). *EcfabH* genes encode 3-ketoacyl-ACP synthase III, the enzyme required for initiation of fatty acid biosynthesis in bacteria [10]. In order to confirm the function of *fabH* in *R*. *solanacearum* fatty acid synthesis, the ability of fatty acid synthesis in strain RsmH was determined by measuring sodium [1-^14^C] acetate incorporation into membrane phospholipids as described in the Materials and Methods section. The RsmH strain synthesized only traces of fatty acids when grown in medium lacking octanoic acid (Fig 2B, **lane 2**). Complementation via episomal expression of *E*. *coli fabH* or *R*. *solanacearum fabH* restored fatty acid biosynthesis in RsmH strain (Fig 2B, **lanes 3 and 4**). These data confirmed that deletion of *fabH* caused octanoic acid auxotrophy in *R*. *solanacearum*, and the *RsfabH*-encoded protein had 3-ketoacyl-ACP synthase III activity.

**Fig 3:**
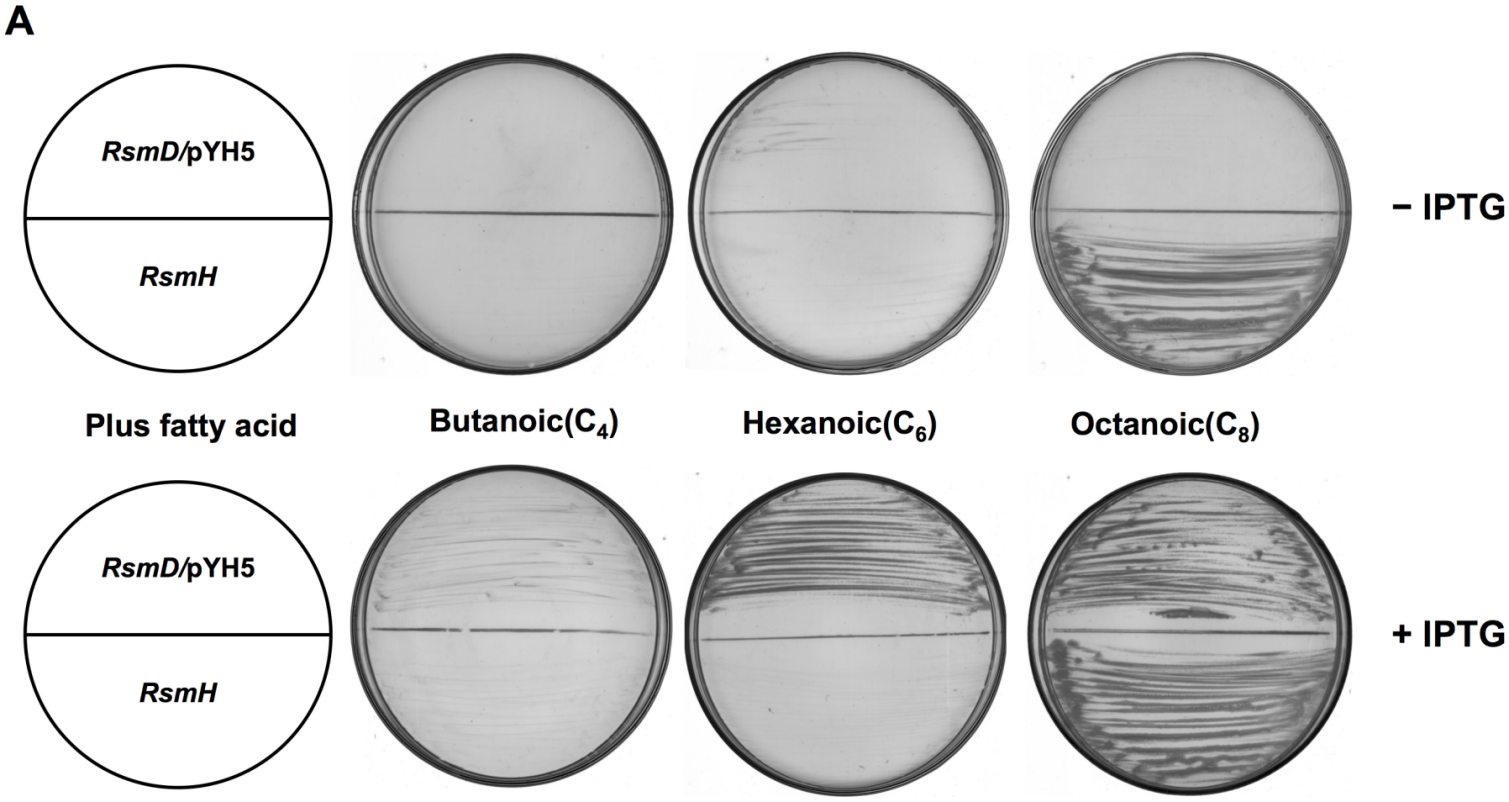
Characterization of *R*. *solanacearum fabH* mutant RsmH and *fabH fabW* double mutant RsmD on BG medium in the presence of various fatty acid species. (A) The growth of mutants on BG medium in the presence of butanoic, hexanoic, or octanoic acid. (B) The growth of mutants on BG medium in the presence decanoic, dodecanoic, or tetradecanoic acid. −IPTG indicates no addition of IPTG to induce the expression of *V*.*harveyi aasS*; +IPTG indicates addition of IPTG to induce the expression of *V*.*harveyi aasS*.

We also tested if other fatty acid species restored RsmH growth on BG medium. Data showed that short-chain fatty acids, such as butanoic (C_4_) or hexanoic (C_6_) acid, failed to restore growth of RsmH, while the long-chain fatty acids, tetradecanoic (C_14_), hexadecanoic (C_16_), or octadecanoic (C_18_) acids supported RsmH growth, but it was much weaker than the growth on BG medium supplied with octanoic (C_8_), decanoic (C_10_), or dodecanoic (C_12_) acids (Fig 3A and 3B). These results suggested that *R*. *solanacearum* possesses another enzyme(s) that allows exogenous fatty acids to enter the fatty acid synthesis pathway, and has a strong preference for fatty acids of medium chain length.

The RsmW strain grew well on BG medium in the absence of octanoic acid (Fig 2A). To determine if *RsfabW* is responsible for growth of RsmH on BG medium in the presence of fatty acids, double deletion of the *RsfabH* and *RsfabW* strains was constructed. Because strains lacking both RsFabH and RsFabW activities might be nonviable, we first introduced plasmid pYH5 that encodes *Vibrio harveyi* acyl-ACP synthetase (AasS) from plasmid pSRK-Gm into RsmH and then deleted *fabW* by allelic replacement. Double deletion mutant RsmHW [*ΔfabH ΔfabW*/pYH5 (pSRK-*aasS*)] was obtained by selection on BG medium containing octanoic acid and IPTG. The double deletion strain RsmD was unable to grow on BG medium in the absence of octanoic acid (Fig 2A). Without IPTG induction, RsmHW was unable to grow on BG medium containing any species of fatty acids (Fig 3A and 3B). However, under the induction by IPTG, RsmD grew on BG medium containing C_4_, C_6_, C_8_, or C_10_ fatty acid, but failed when the supplement was long chain fatty acids, such as C_12_, C_14_, C_16_, or C_18_ fatty acids (Fig 3A and 3B **)**. The lack of growth on BG medium containing fatty acids in the absence of IPTG induction indicated that double mutant RsmHW lost both RsFabH and RsFabW activities and failed to synthesize fatty acids, whereas growth on BG medium containing short or medium chain fatty acid under IPTG induction indicated that the presence of acyl-ACP synthetase (AasS) activity, and RsmHW allows exogenous fatty acids to enter *R*. *solanacearum* and restore fatty acid synthesis. These results confirmed that *fabW* is responsible for growth of strain RsmH on BG medium in the presence of fatty acids, and confirmed that *RsfabW* encodes a 3-ketoacyl-ACP synthase III similar to *P*. *aeruginosa* PA3286 that is able to condense malonyl-ACP with medium-chain acyl-CoAs to initiate fatty acid biosynthesis.

### Expression and purification of the *R*. *solanacearum* RsFabH and RsFabW

In order to allow direct *in vitro* assays of RsFabH and RsFabW KAS activities, both proteins were expressed in *E*. *coli* and the N-terminally His_6_-tagged versions of these two proteins were successfully purified by nickel-chelate chromatography (S1A Fig). The purified RsFabH and RsFabW proteins had monomeric molecular masses of 36 kDa and 42 kDa on SDS-PAGE, respectively, which were in agreement with the values calculated from the sequences of the tagged proteins (36.6 and 42.4 kDa, respectively). We also purified the *E*. *coli* fatty acid biosynthetic proteins FabD, FabG, FabZ, FabI, and holo-ACP, plus the *V*. *harveyi* acyl-ACP synthetase (AasS) (see Materials and Methods).

### In *vitro* enzymatic activities of RsFabH and RsFabW

To determine the function of RsFabH, the initiation reaction of the fatty acid synthesis were reconstituted by the addition of EcFabD, 3-ketoacyl-ACP synthase III (EcFabH or RsFabH), EcFabG, EcFabA, and EcFabI, and the products was analyzed by conformationally sensitive gel electrophoresis. In the absence of KAS, only holo-ACP was observed during electrophoresis. Addition of EcFabH or RsFabH to the reaction mixture led to formation of butyryl-ACP (S1B Fig, **lane 1 and lane 2**). Upon addition of the long chain *E*. *coli* 3-ketoacyl-ACP synthase I, FabB, to the reactions, all reactions produced long chain acyl-ACP species (S1B Fig, **lane 3 and lane 4**). These data clearly showed that, like *E*. *coli* FabH, RsFabH could complete the initial cycle of fatty acid synthesis to produce butyryl-ACP.

**Fig 4:**
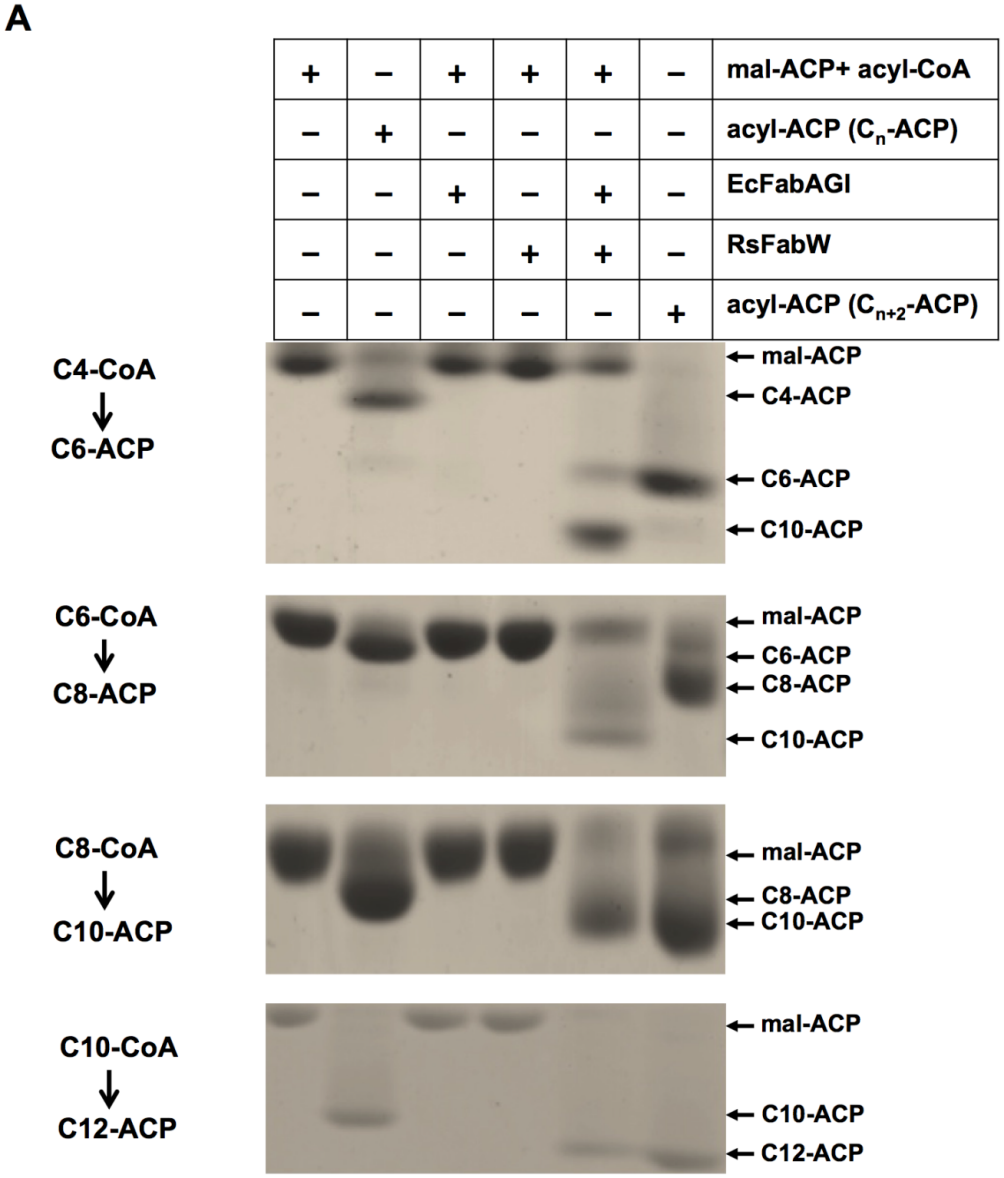
Condensation of malonyl-ACP with acyl-CoAs (C_2_-CoA to C_10_-CoA) or acyl-ACPs (C_4_-ACP to C_10_-ACP) by purified *R*. *solanacearum* FabW. (A) The cycle of fatty acid synthesis was reconstructed *in vitro* using a combination of *E*. *coli* FabA, FabG, FabI, and *R*. *solanacearum* FabW with NADH, and NADPH as cofactors, and malonyl-ACP plus acyl-CoAs (C_4_-CoA to C_10_-CoA) as substrates. (B) The reaction mixture of fatty acid synthesis contained *E*. *coli* FabA, FabG, FabI, and *R*. *solanacearum* FabW or *E*. *coli* FabB, NADH, and NADPH as cofactors, and malonyl-ACP plus acyl-ACPs (C_6_-ACP to C_10_-ACP) as substrates. (C) Condensation of malonyl-ACP with acetyl-CoA or butyryl-ACP catalyzed by *R*. *solanacearum* FabW. The initial reaction of fatty acid synthesis was reconstructed using a combination of *E*. *coli* FabD, FabA, FabG, FabI, and *R*. *solanacearum* FabH (lane 2) or FabW (lane 3) with NADH, and NADPH as cofactors and malonyl-CoA and acetyl-CoA as substrates. The elongation reaction of fatty acid synthesis was reconstructed by addition of *E*. *coli* FabB (lane 4) or *R*. *solanacearum* FabW (lane 5) to the initial reaction catalyzed by *R*. *solanacearum* FabH.

To confirm the functions of RsFabW, which condenses malonyl-ACP with medium-chain acyl-CoAs to produce 3-keto-acyl-ACPs, we tested condensation of malonyl-ACP with octanoyl-CoA. First, malonyl-ACP was synthesized from holo-ACP and malonyl-CoA using *E*. *coli* FabD. Then, the reactions were reconstituted by the sequential addition of purified proteins and co-factors as described in the Materials and Methods section. After incubation of *E*. *coli* FabG, FabA, and FabI with malonyl-ACP, octanoyl-CoA, NADPH, and NADH, the reaction mixture did not form new products, and incubation of RsFabW with only malonyl-ACP, octanoyl-CoA, NADPH, and NADH, also failed to produce any new species. However, when RsFabW was added to the mixture of *E*. *coli* FabG, FabA, and FabI with malonyl-ACP, octanoyl-CoA, NADPH, and NADH, then decanoyl-ACP was formed (Fig 4A). Using the same approach, decanoyl-CoA was tested and the result showed that, like octanoyl-CoA, decanoyl-CoA was also one of the substrates necessary for RsFabW to convert dodecanoyl-ACP (Fig 4A). We also tested butyryl-CoA and hexanoyl-CoA. Although both butyryl-CoA and hexanoyl-CoA were substrates of RsFabW, the product of the reaction was neither hexanoyl-ACP nor octanoyl-ACP. It was surprising that butyryl-CoA and hexanoyl-CoA were converted to decanoyl-ACP by RsFabW (Fig 4A). This suggested that RsFabW was able to condense acyl-ACPs and malonyl-ACP to produce longer acyl-ACPs. To confirm this possibility, we replaced octanoyl-CoA with octanoyl-ACP to perform the same reaction. Indeed, the combination of RsFabW, *E*. *coli* FabG, FabA, and FabI, in the reaction mixture produced decanoyl-ACP (Fig 4B), and hexanoyl-ACP gave the same product, decanoyl-ACP (Fig 4B). However, when decanoyl-ACP was tested, no new products appeared (Fig 4B). Elongation of butyryl-ACP by RsFabW was also tested. Because AasS does not use butyric acid, butyryl-ACP was produced using the initial reaction of the fatty acid synthesis reconstituted by the sequential addition of *E*. *coli* FabD, FabH, FabA, FabG, and FabI, and malonyl-CoA, holo-ACP, NADPH, and NADH (Fig 4C, **lane 2**). Upon addition of RsFabW, the reaction mixture produced hexanoyl-ACP and decanoyl-ACP (Fig 4C, **lane 5**). Thus, these data indicate that RsFabW is able to use butyryl-ACP, hexanoyl-ACP, and octanoyl-ACP as substrates and to elongate them to decanoyl-ACP.

**Fig 5:**
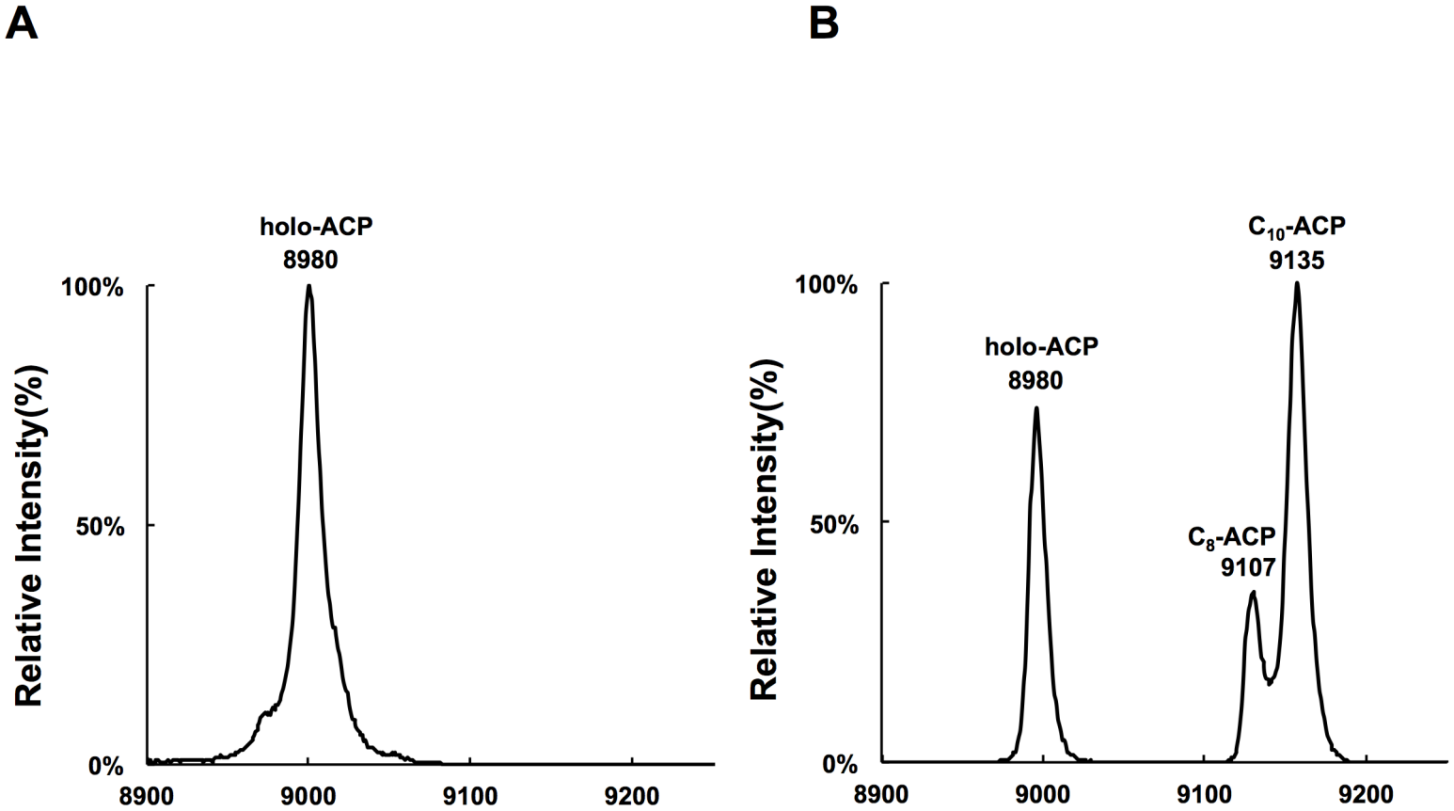
MALDI-TOF-MS of the products of the RsFabW reaction. (A) Mass spectrum of the reaction mixture not containing RsFabW. (B) Mass spectrum of the reaction mixture containing RsFabW.

The above ACP thioester intermediates in the reconstitution assays were also analyzed by MALDI-TOF-MS to confirm the identities of the products. We used hexanoyl-CoA as substrate and purified the products of the reaction mixture before measuring their mass by MALDI-TOF-MS. As a control (Fig 5A), the mass peak for the reaction not containing RsFabW occurred at 8980, corresponding to the holo-ACP molecules. For RsFabW-containing reaction mixtures, two new peaks appeared. The minor mass peak was at 9107, corresponding to octanoyl-ACP (holo-ACP + 128), and the major peak was at 9135, corresponding to decanoyl-ACP (holo-ACP + 155) (Fig 5B). These data confirm the identities determined by gel electrophoresis as indicated in Fig 4A and 4B, and substantiate the conclusion that RsFabW was able to condense either medium-chain acyl-CoAs or medium-chain acyl-ACPs with malonyl-ACP.

We next determined if RsFabW could condense acetyl-CoA with malonyl-ACP to initiate fatty acid synthesis. The reaction mixture contained *E*. *coli* FabD, FabA, FabG, and FabI, malonyl-CoA, acetyl-CoA, holo-ACP, NADPH, and NADH. When adding RsFabW to the mixture and incubating for 1 hour, the product was assayed by conformationally sensitive gel electrophoresis, showing that butyryl-ACP, hexanoyl-ACP, and decanoyl-ACP were formed (Fig 4C, **lane 3**). However, when adding *R*. *solanacearum* FabH to the mixture, only butyryl-ACP was produced (Fig 4C, **lane 2**). In conclusion, *in vitro*, RsFabW is able to use acetyl-CoA to initiate fatty acid synthesis and then produce decanoyl-ACP.

We also compared the 3-ketoacyl-ACP synthase III activities of cell-free extracts of wild type strain GMI1000 and the *ΔfabH* (RsmH) or *ΔfabW* (RsmW) deletion mutants (S2A Fig). The cell-free extracts of the wild type strain GMI1000 could use acyl-CoAs (C_2_-CoA to C_10_-CoA) as substrates to synthesize long-chain acyl-ACP. In the *ΔfabW* mutant extract, only acetyl-CoA was elongated to long-chain acyl-ACPs, whereas the *ΔfabH* mutant extract elongated acyl-CoAs (C_4_-CoA to C_10_-CoA) to long-chain acyl-ACP species. The double mutant RsmD extract failed to elongate any of the acyl-CoAs.

### The substrate specificity of RsFabW

In the above *in vitro* experiments, it has been demonstrated that RsFabW is able to use acyl-CoAs (C_2_ to C_10_) as primers to initiate fatty acid synthesis. In order to probe the substrate specificity of RsFabW, the decrease in absorbance at 340 nm of NADPH was monitored spectrophotometrically in reaction mixtures containing holo-ACP, malonyl-CoA, NADPH, EcFabD, EcFabG, RsFabW, and various straight-chain saturated acyl-CoAs (C_2_ to C_10_) (Table 1). The data showed that although the conformationally sensitive gel assay indicated that RsFabW could elongate acetyl-CoA to decanoyl-CoA, RsFabW very weakly accepted acetyl-CoA as the primer; the activity for acetyl-CoA was 16.93 ± 0.83 μmol/m × μg. The best substrate was clearly octanoyl-CoA, and the activity of RsFabW for octanoyl-CoA was 195.77 ± 14.55 μmol/s × μg. For butyryl-CoA, hexanoyl-CoA, and decanoyl-CoA, the activities of RsFabW were 19.05 ± 1.61, 77.38 ± 6.06, and 97.88 ± 5.4 μmol/s×μg, respectively, and lower than that for C_8_-CoA, confirming the observations that the shorter exogenous fatty acids, such as butyric and hexanoic acid, did not rescue growth in *ΔfabH* strain.

**Table 1:** Substrate specificities of *Ralstonia solanacearum* FabH and FabW.

Substrates	Enzyme activity (μmol/sec/μg) (mean ± SD)	
	RsFabH	RsFabW
Acetyl-CoA	145 ± 6.33	< 1
Butyryl-CoA	< 1	19.05 ±1.61
Hexanoyl-CoA	< 1	77.38 ± 6.06
Octanoyl-CoA	< 1	195.77 ± 14.55
Decanoyl-CoA	< 1	97.88 ± 5.4

To confirm that RsFabW uses butyryl-CoA and hexanoyl-CoA as substrates *in vivo*, we introduced plasmid pYH6 (pSRK-*RsfabW*) into the RsmH strain to construct the *RsfabW* overexpression strain RsmH/pYH6 (pSRK-*RsfabW*), and tested the growth of this strain on BG plates in the presence of acetic, butanoic, or hexanoic acid. Under IPTG induction strain RsmH/pYH6 was able to grow on BG plates containing butanoic or hexanoic acid, but still failed to grow on BG containing acetic acid, though the growth of RsmH/pYH6 was weak on BG containing butanoic acid (S2B Fig). This indicated that *in vivo*, RsFabW uses butyryl-CoA and hexanoyl-CoA as substrates.

## Discussion

In general, the 3-ketoacyl-ACP synthase III class of enzymes condenses acyl-CoAs with malonyl-ACP to initiate fatty acid biosynthesis in the dissociated, type II fatty acid synthase systems typified by bacteria [1,3,10,11]. According to the substrate specificity of enzymes, the 3-ketoacyl-ACP synthase III class of enzymes can be divided into three groups. The first group of these enzymes that is highly selective for acetyl-CoA as primers includes *E*. *coli* FabH [33] and *P*. *aeruginosa* FabY [17]. The second group of these enzymes includes many gram-positive bacteria FabHs, such as *L*. *monocytogenes* [15] and *B*. *subtilis* [34], which prefer branched-chain acyl-CoAs from branched-chain amino acid metabolism as substrates. The third group contains *M*. *tuberculosis* FabH [10], and *P*. *aeruginosa* PA3286 [9], which uses medium-chain acyl-CoAs as substrates and shunt intermediates from the type I fatty acid synthesis pathway (type I FAS) or the fatty acid β-oxidation degradation cycle into the type II fatty acid synthesis pathway. The *R*. *solanacearum* genome encodes two forms of 3-ketoacyl-ACP synthase III [19]. RsFabH catalyzes the condensation of acetyl-CoA with malonyl-ACP in initiation steps of fatty acid biosynthesis, and the RsmH mutant strain has lost the ability for *de novo* fatty acid synthesis. RsFabW can condense either acyl-CoAs (C_2_ to C_10_-CoAs) or acyl-ACPs (C_4_ to C_8_-ACPs) with malonyl-ACP to produce 3-keto-acyl-ACP *in vitro*. Moreover, although the RsmW mutant was viable, *RsfabW* was responsible for *RsfabH* mutant growth on medium containing medium-chain fatty acids, such as octanoic, decanoic, and dodecanoic acid. These results confirmed that *R*. *solanacearum* not only utilizes acetyl-CoA, but also medium-chain acyl-CoAs or acyl-ACPs as primers to initiate fatty acid synthesis.

**Fig 6:**
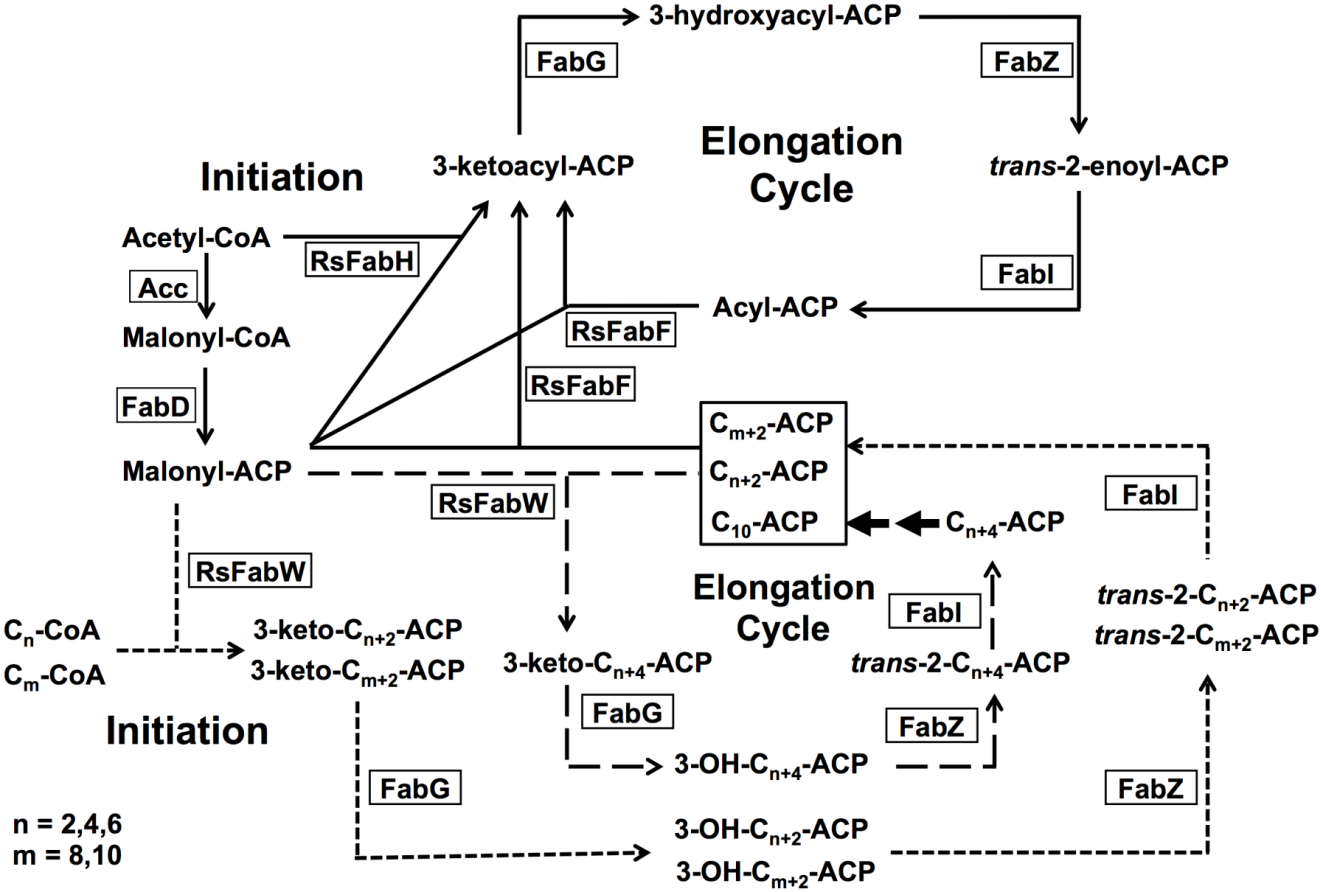
Model of the fatty acid biosynthesis pathway in *R*. *solanacearum*. Abbreviations: Acc, acetyl-CoA carboxylase; FabD, malonyl-CoA: ACP transacylase; RsFabH, 3-ketoacyl-ACP synthase III; RsFabW, 3-ketoacyl-ACP synthase III; FabG, 3-ketoacyl-ACP reductase; FabZ, 3-hydroxyacyl-ACP dehydratase; RsFabF, 3-ketoacyl-ACP synthase II; FabI, enoyl-ACP reductase. The full line indicates RsFabH uses acetyl-CoA substrate to initiate the fatty acid synthesis. The dotted line indicates RsFabW uses acyl-CoAs as substrates to initiate the fatty acid synthesis. The dashed line indicates RsFabW uses acyl-ACPs as substrates to initiate the fatty acid synthesis.

The growth of *RsfabH* mutants on medium containing long-chain fatty acids (C_14_ to C_18_) was weaker than on medium in the presence of medium-chain fatty acids (C_8_ to C_12_) (Fig 3). This was in agreement with the substrate specificity of RsFabW for C_8_-CoA and C_10_-CoA *in vitro* (Table 1). This indicated that exogenous long-chain fatty acids (C_14_ to C_18_) should be degraded to C_8_-CoA by β-oxidation, and then RsFabW would shunt C_8_-CoA into the fatty acid synthesis pathway to make LPS, UFA, and SFA required for bacteria growth. However, although FabW possessed strong sequence similarity to *P*. *aeruginosa* PA3286 (Fig 1D), FabW was different from *P*. *aeruginosa* PA3286 [9]. First, FabW not only uses acyl-CoAs (such as octanoyl-CoA or decanoyl-CoA) as primers, but also condenses short-chain acyl-ACPs (such as butanoyl-ACP, hexanoyl-ACP, and octanoyl-ACP) with malonyl-ACP. Second, FabW seems to have a unique role in fatty acid synthesis in *R*. *solanacearum*, but does not only shunt intermediates from β-oxidation degradation into fatty acid biosynthesis. *R*. *solanacearum* mainly invades xylem vessels of host plants [35], where this is a lack of sufficient phospholipids or free fatty acids to support this bacterial growth. RsFabW is also distinct from long-chain 3-ketoacyl-ACP synthase I/II, which can catalyze the condensation of long-chain acyl-ACP with malonyl-ACP in the elongation cycle of bacterial fatty acid synthesis [10]. RsFabW did not utilize decanoyl-ACP or longer acyl-ACPs as substrates. It has been demonstrated that *R*. *solanacearum* only has one long chain 3-ketoacyl-acyl carrier protein synthase, RsFabF1, which possesses both the activity of 3-ketoacyl-ACP synthase II and I [23]. Therefore, it is possible that RsFabW helps RsFabF1 to function in elongation reactions of medium-chain fatty acid synthesis. On the basis of our results, we suggest a model for fatty acid biosynthesis in *R*. *solanacearum* in Fig 6.

Although *V*. *harveyi aasS* was able to bypass both the *E*. *coli fabH* mutation [29] and *R*. *solanacearum ΔfabH ΔfabW* double mutation when medium was supplemented with exogenous short-chain fatty acids, such as hexanoic or octanoic acid, the *R*. *solanacearum ΔfabH ΔfabW* double mutant is different from the *E*. *coli fabH* mutant. Decanoic acid did not support *E*. *coli fabH* carried the *V*. *harveyi aasS*-encoded plasmid for growth [29], but actually did maintain the *ΔfabH ΔfabW* double mutant that harbored *aasS* encoded plasmid for growth, though the growth was weaker than that observed with supplementation with hexanoic or octanoic acid. The growth of the *E*. *coli fabH* mutant carrying *aasS*-encoding plasmid was not supported by decanoic acid and this was because decanoyl-ACP only entered the fatty acid synthesis pathway beyond the point where unsaturated fatty acid synthesis branches from the common (saturated) pathway [29]. However, to date it is still not clear how *R*. *solanacearum* synthesizes unsaturated fatty acids. One possibility is that *R*. *solanacearum* converted decanoyl-ACP to *cis*-3-decenoyl-ACP directly, which is the key intermediate for unsaturated fatty acids synthesis. BLAST analysis of the *R*. *solanacearum* GMI1000 genome showed that RSc 0114 is 55% identical to *Neisseria gonorrhoeae* UfaA, which has been demonstrated to function in anaerobic unsaturated fatty acid synthesis[36]. Although *R*. *solanacearum* is an aerobic bacterium, whether or not *R*. *solanacearum* RSc0114 could convert decanoyl-ACP to *cis*-3-decenoyl-ACP directly needs to be further addressed.

## Supporting Information

S1 Fig Purification of *R*. *solanacearum* FabH and FabW from *E*. *coli* strain BL21 (DE3) and the function of *R*. *solanacearum* FabH in the initial cycle of fatty acid synthesis *in vitro*. (A) Purification of *R*. *solanacearum* FabH and FabW by native nickel-chelate chromatography. Lane 1, molecular mass markers; lane 2, *R*. *solanacearum* FabH; lane 3, *E*. *coli* FabH; lane 4, *R*. *solanacearum* FabW. (B) The initial cycle of fatty acid synthesis was reconstructed *in vitro* using a combination of *E*. *coli* FabD, KAS (*E*. *coli* FabH (EcH) (lane 1), or *R*. *solanacearum* FabH (RsH) (lane 2)), *E*. *coli* FabG (EcG), *E*. *coli* FabA (EcA), and FabI (EcI) enzymes, ACP, NADH, and NADPH as cofactors, and malonyl-ACP plus acetyl-CoA as substrates to produce butyryl-ACP. To complete the fatty acid synthesis reaction (lanes 3 to 4), *E*. *coli* FabB (EcB) was added to the reactions.
(TIFF)

S2 Fig Analysis of the activities of fatty acid synthesis in cell-free extracts of *R*. *solanacearum* mutants and the growth of strain Δ*fabH*/pYH6 on BG plates in the presence of short-chain fatty acids. (A) Analysis of the activities of fatty acid synthesis in cell-free extracts of *R*. *solanacearum* mutants. WT, indicates the cell-free extracts of *R*. *solanacearum* GMI1000; Δ*fabW*, indicates the cell-free extracts of *R*. *solanacearum fabW* mutant strain RsmW; Δ*fabH*, indicates the cell-free extracts of *R*. *solanacearum fabH* mutant strain RsmH; Δ*fabH* Δ*fabW*, indicates the cell-free extracts of *R*. *solanacearum fabH fabW* double mutant strain RsmD. Abbreviations: C_2_-CoA, acetyl-CoA; C_4_-CoA, butyryl-CoA; C_6_-CoA, hexanoyl-CoA; C_8_-CoA, octanoyl-CoA; C_10_-CoA, decanoyl-CoA; C_12_-CoA, dodecanoyl-CoA. (B) The growth of strain Δ*fabH*/pYH6 on BG plates in the presence of short-chain fatty acids. The strain Δ*fabH*/pYH6 carried *RsfabW* encoded plasmids and grew on BG plates containing various fatty acids under IPTG induction.
(TIFF)

S1 Table The strains and plasmids used in this study. (DOCX)

S2 Table Sequences of the PCR primers used in this study. (DOCX)
