# Supplementary figures and images for: Ralstonia solanacearum RSp0194 Encodes a Novel 3-Keto-Acyl Carrier Protein Synthase III
Source: PLoS One. 2015 Aug 25;10(8):e0136261. doi: 10.1371/journal.pone.0136261 (PMC4549310; doi:10.1371/journal.pone.0136261)

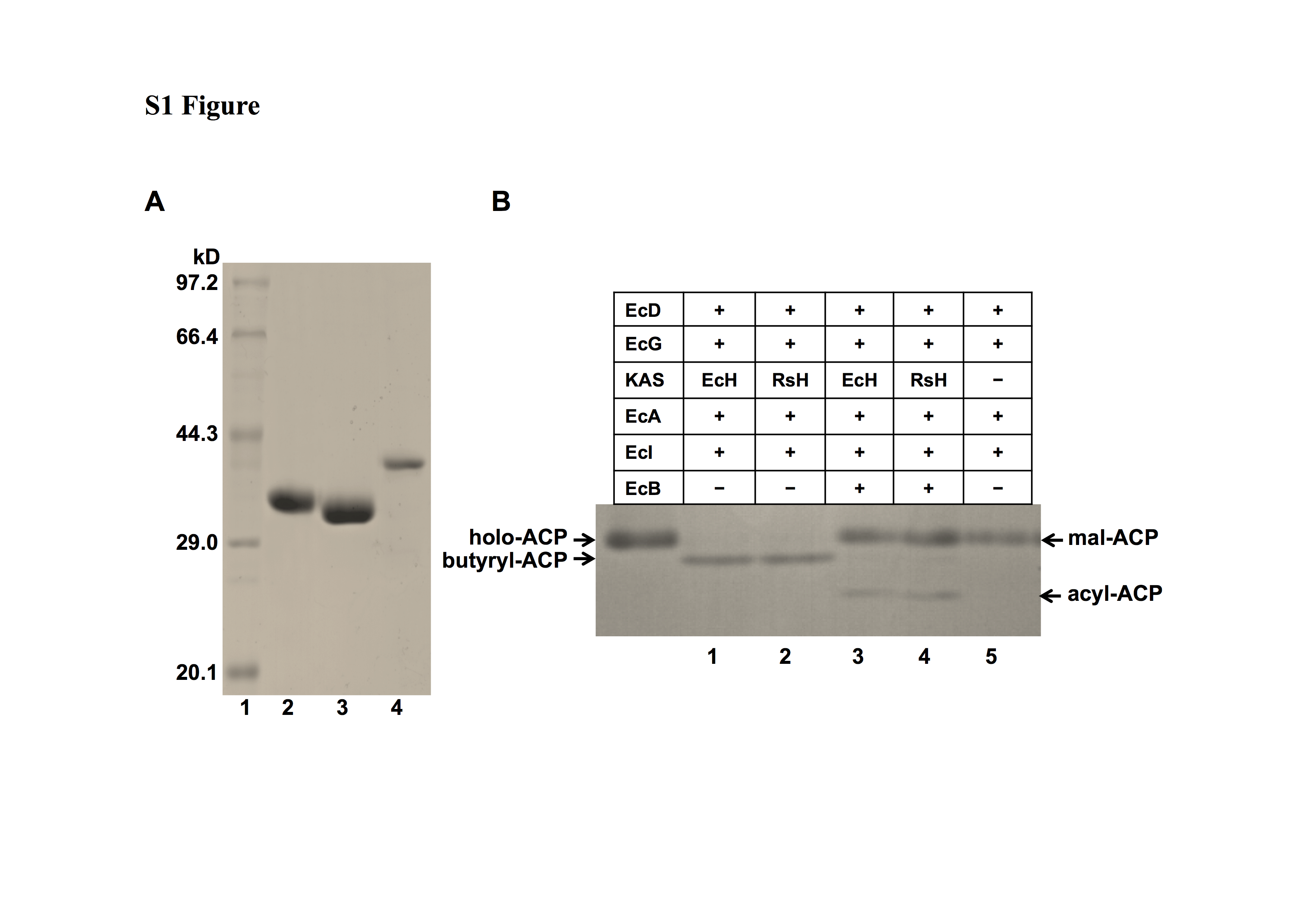

Supplement: S1 Fig — (A) Purification of R. solanacearum FabH and FabW by native nickel-chelate chromatography. Lane 1, molecular mass markers; lane 2, R. solanacearum FabH; lane 3, E. coli FabH; lane 4, R. solanacearum FabW. (B) The initial cycle of fatty acid synthesis was reconstructed in vitro using a combination of E. coli FabD, KAS (E. coli FabH (EcH) (lane 1), or R. solanacearum FabH (RsH) (lane 2)), E. coli FabG (EcG), E. coli FabA (EcA), and FabI (EcI) enzymes, ACP, NADH, and NADPH as cofactors, and malonyl-ACP plus acetyl-CoA as substrates to produce butyryl-ACP. To complete the fatty acid synthesis reaction (lanes 3 to 4), E. coli FabB (EcB) was added to the reactions. (TIFF) [file pone.0136261.s001.tiff]
