# Supplementary material for: Ralstonia solanacearum RSp0194 Encodes a Novel 3-Keto-Acyl Carrier Protein Synthase III
Source: PLoS One. 2015 Aug 25;10(8):e0136261. doi: 10.1371/journal.pone.0136261 (PMC4549310; doi:10.1371/journal.pone.0136261)
Supplement: S1 Table — (DOCX) [file pone.0136261.s003.docx]

**S1 Table. The strains and plasmids used in this study.**

| **Strains/Plasmids** | | **Relevant genotype or characteristics** | **Sources** |
| --- | --- | --- | --- |
| ***E. coli*** | | | |
| **DH5α** | F^-^*deoR* *endA1* *gyrA96* *hsdR17* (r_K_^-^m_K_^+^) *recA1* *relA1* *supE44* *thi-1*Δ (*lacZYA-argF*) *U169*(ϕ80*lacZ*ΔM15) | | NBRP |
| **BL21(DE3)** | F^-^ *dcm* *ompT hsdS*(r_B_^-^ m_B_^-^) *gal* (λDE3) | | NBRP |
| **S17-1** | F^-^ *thi* *pro* *hsdR* [RP4-2 Tc::Mu Km::Tn7 (Tp Sm)] | | NBRP |
| ***R. solanacearum*** | | | |
| **GMI1000** | Wild-type strain | | ATCC |
| **RsmH** | GMI1000 Δ*fabH* | | This work |
| **RsmW** | GMI1000 Δ*fabW* | | This work |
| **RsmHW** | GMI1000 Δ*fabH*Δ*fabW* strain carrying *Vibrio harveyi* *aasS*-encoding plasmid, pYH5 | | This work |
| **YH4** | RsmH /pYH3 | | This work |
| **YH5** | RsmW /pYH4 | | This work |
| **Plasmids** | | | |
| **pMD19** | Amp^r^; T-vector | | Takara |
| **pBAD24M** | Amp^r^; expression vector | | [28] |
| **pET-28 (b)** | Km^r^; expression vector | | Novagen |
| **pK18mobscaB** | Km^r^; *sacB*-based gene replacement vector | | [25] |
| **pSRK-Gm** | Gm^r^; pBBR1MCS-2-derived broad-host-range expression vector containing the *lac* promoters, *lacI^q^* and *lacZα*^+^ | | [24] |
| **pYH1** | Amp^r^; *R. solanacearum fabH* in pMD19-T | | This work |
| **pYH2** | Amp^r^; *Escherichia coli fabH* in pMD19-T | | This work |
| **pYH12** | Amp^r^; *R. solanacearum fabW* in pMD19-T | | This work |
| **pYH3** | Gm^r^; *RsfabH* cloned between the NdeI and HindIII sites of pSRK-Gm | | This work |
| **pYH4** | Gm^r^; *EcfabH* from pYH2 digested with NdeI and EcoRI and inserted into the same sites of pSRK-Gm | | This work |
| **pYH5** | Gm^r^; *V. harveyi aaS* cloned between the NdeI and HindIII sites of pSRK-Gm | | This work |
| **pYH6** | Gm^r^; *RsfabW* cloned between the NdeI and HindIII sites of pSRK-Gm | | This work |
| **pYH7** | Km^r^; *RsfabH* from pYH1 digested with NdeI and HindIII and inserted into the same sites of pET28 (b) | | This work |
| **pYH8** | Km^r^; Ec*fabH* in pET28 (b) (constructed as for pYH7) | | This work |
| **pYH9** | Km^r^; *fabW* in pET28(b) (constructed as for pYH7) | | This work |
| **pYH10** | Km^r^; pK18mobscaB carrying *RsfabH* deletion fragment | | This work |
| **pYH11** | Km^r^; pK18mobscaB carrying carrying *RsfabW* deletion fragment | | This work |
