# Supplementary material for: Ralstonia solanacearum RSp0194 Encodes a Novel 3-Keto-Acyl Carrier Protein Synthase III
Source: PLoS One. 2015 Aug 25;10(8):e0136261. doi: 10.1371/journal.pone.0136261 (PMC4549310; doi:10.1371/journal.pone.0136261)
Supplement: S2 Table — (DOCX) [file pone.0136261.s004.docx]

**S2 Table. Sequences of the PCR primers used in this study.**

| Primer name | Primer sequence (5′ to 3′) | Digestion sites ^α^ |
| --- | --- | --- |
| RsfabH NdeI | CTACATATGACACGTTACGCAAGGATC | NdeI |
| RsfabH HindIII | CATAAGCTTTCAGAAGCGCAGCAACGC | HindIII |
| Rsp0194 NdeI | GTTACGCATATGCATGATGTCGTCATC | NdeI |
| Rsp0194 BamHI | ATAGGATCCTCAGCGCTTGCGCACC | BamHI |
| RsfabH Up EcoRI | CAGGAATTCAAGAGGTCATCAAGGGCAAC | EcoRI |
| RsfabH Up XbaI | CTATCTAGAGTAACGTGTCATGTGTGATG | XbaI |
| RsfabH Dn XbaI | TGACACGTTACTCTAGATAGGATGAGCACGGCAACACGTC | XbaI |
| RsfabH Dn HindIII | CTAAAGATTCATTGAAATTGACGGCCTCC | HindIII |
| fabH HW-1 | TGTCGGTGGTCGAACACAAG |  |
| fabH HW-2 | CCCTTAGCCTTGGCGATCTC |  |
| Rsp0194 Up EcoRI | ACTGAATTCGCAATCGACATGGCGTACG | EcoRI |
| Rsp0194 Up | AGCGTACGTGCGTGAAATTGGC |  |
| Rsp0194 Dn | CAATTTCACGCACGTACGCTCCGGACGGTCGCGGGCGG |  |
| Rsp0194 Dn BamHI | ATCGGATCCACGAACTTTGTCCACGAGC | BamHI |
| Rsp0194 HW-1 | ATCCTGGACCGGCTGATC |  |
| Rsp0194 HW-2 | GAAGCGAAGCCTGCCATC |  |

*^a^*Underlined nucleotide sequences are digestion sites of restriction endonuclease.
